# Differential regulation of flower transpiration during abiotic stress in annual plants

**DOI:** 10.1111/nph.18162

**Published:** 2022-05-12

**Authors:** Ranjita Sinha, Sara I. Zandalinas, Yosef Fichman, Sidharth Sen, Shuai Zeng, Aurelio Gómez‐Cadenas, Trupti Joshi, Felix B. Fritschi, Ron Mittler

**Affiliations:** ^1^ Division of Plant Sciences and Technology College of Agriculture Food and Natural Resources and Interdisciplinary Plant Group University of Missouri Columbia MO 65211 USA; ^2^ Departamento de Ciencias Agrarias y del Medio Natural Universitat Jaume I Castelló de la Plana 12071 Spain; ^3^ Institute for Data Science and Informatics and Interdisciplinary Plant Group University of Missouri Columbia MO 65211 USA; ^4^ Department of Electrical Engineering and Computer Science University of Missouri Columbia MO 65211 USA; ^5^ Department of Health Management and Informatics, and Christopher S. Bond Life Sciences Center University of Missouri Columbia MO 65211 USA; ^6^ Department of Surgery University of Missouri School of Medicine Christopher S. Bond Life Sciences Center University of Missouri Columbia MO 65201 USA

**Keywords:** climate change, drought, heat stress, soybean, stomata, stress combination, transpiration, yield

## Abstract

- Heat waves occurring during droughts can have a devastating impact on yield, especially if they happen during the flowering and seed set stages of the crop cycle. Global warming and climate change are driving an alarming increase in the frequency and intensity of combined drought and heat stress episodes, critically threatening global food security.
- Because high temperature is detrimental to reproductive processes, essential for plant yield, we measured the inner temperature, transpiration, sepal stomatal aperture, hormone concentrations and transcriptomic response of closed soybean flowers developing on plants subjected to a combination of drought and heat stress.
- Here, we report that, during a combination of drought and heat stress, soybean plants prioritize transpiration through flowers over transpiration through leaves by opening their flower stomata, while keeping their leaf stomata closed. This acclimation strategy, termed ‘differential transpiration’, lowers flower inner temperature by about 2–3°C, protecting reproductive processes at the expense of vegetative tissues.
- Manipulating stomatal regulation, stomatal size and/or stomatal density of flowers could serve as a viable strategy to enhance the yield of different crops and mitigate some of the current and future impacts of global warming and climate change on agriculture.

## Introduction

The unyielding increase in atmospheric and oceanic temperatures, termed ‘global warming’, is causing drastic changes in our climate, termed ‘climate change’ (Lobell *et al*., 2011; Steg, 2018; Bailey‐Serres *et al*., 2019; Alizadeh *et al*., 2020; Overpeck & Udall, 2020; von der Gathen *et al*., 2021; Zandalinas *et al*., 2021; Zhai *et al*., 2021). As a result, large areas of our planet are increasingly exposed to floods or extended droughts combined with extreme temperatures (Mazdiyasni & AghaKouchak, 2015; Alizadeh *et al*., 2020; Overpeck & Udall, 2020; Rivero *et al*., 2022; Zandalinas *et al*., 2021). Historically, extended droughts combined with heat waves have been the cause of catastrophic reductions in agricultural productivity estimated at billions of dollars per episode (e.g. the drought and heat wave combination events that occurred during the summers of 1980 and 1988 in the US resulted in losses to agriculture estimated at $33 and 44 billion, respectively; https://www.ncdc.noaa.gov/billions/; Mittler, 2006; Lobell *et al*., 2011; Rivero *et al*., 2022). Because global warming and climate change are increasing the frequency and intensity of drought and heat stress combination events worldwide, more studies are needed to understand how crops and other plants respond to this type of stress combination (Mazdiyasni & AghaKouchak, 2015; Alizadeh *et al*., 2020; Rivero *et al*., 2022; Zandalinas *et al*., 2021; Zhai *et al*., 2021). Agricultural experience, as well as multiple studies conducted with different crops, revealed that the effects of drought and heat stress combination on yield of many major grain crops is most severe when the stress combination occurs during the reproductive stage of plant growth (Rollins *et al*., 2013; Mahrookashani *et al*., 2017; Lawas *et al*., 2018; Liu *et al*., 2020; Cohen *et al*., 2021b; Rivero *et al*., 2022; Sinha *et al*., 2021).

Plant reproduction, that is, the developmental process of flower organs (including stamens and stigma), the maturation of pollen and egg cells, pollen shedding, interactions with stigma, germination, growth and eventually fertilization, as well as embryo development and seed filling, are all highly sensitive to elevated temperatures (Santiago & Sharkey, 2019; Djanaguiraman *et al*., 2020; Chaturvedi *et al*., 2021; Sze *et al*., 2021). It was recently proposed that the tightly synchronized nature of the developmental programs involved in these processes, as well as their reliance on certain stress‐related programs (e.g. the stress‐associated dehydration program of pollen grains), reactive oxygen species (ROS) and hormone signaling, under nonstress conditions, makes them more sensitive to stress (Sinha *et al*., 2021; Sze *et al*., 2021). Stresses such as drought or heat, or their combinations, may therefore disrupt these tightly synchronized processes by triggering the activation of stress programs and/or the accumulation of different hormones, ROS or other signals, out of sync with the proper developmental process, leading to the production of immature or malnourished pollen, egg cell programmed cell death and other disruptive processes that decrease yield (Martin *et al*., 2013; Lassig *et al*., 2014; Daneva *et al*., 2016; Zhang *et al*., 2020; Sinha *et al*., 2021; Sze *et al*., 2021).

Many important grain crops such as wheat (*Triticum aestivum*), rice (*Oryza sativa*) and soybean (*Glycine max*) are self‐pollinating and do not require vectors such as insects or wind for cross‐pollination (Liu *et al*., 2006). In many legumes and important grass species, self‐pollination occurs even before the flower opens (i.e. the pollen is transferred to the stigma of the same flower within the closed flower – termed cleistogamy or pseudocleistogamy; Campbell *et al*., 1983; Takahashi *et al*., 2001). Although, under controlled nonstressed conditions, cleistogamy/pseudocleistogamy protects many aspects of the reproductive process from external factors such as low humidity, UV radiation, pathogens and/or other potential stressors, under conditions of drought, or drought combined with heat stress, when transpiration is suppressed, the internal temperature of the flower could rise to high values that would inhibit reproduction (Lawas *et al*., 2018; Wei *et al*., 2018; Sinha *et al*., 2021).

Transpiration in plants is primarily controlled by changes in stomatal aperture and the water vapor pressure differential between the plant and the atmosphere (Will *et al*., 2013; Lawson & Matthews, 2020). When stomata are open, transpiration can occur at a higher rate and cool the plant. This was demonstrated for leaves of different plants subjected to heat stress (Zhou *et al*., 2015; Balfagón *et al*., 2019; Zandalinas *et al*., 2020a). By contrast, during drought, stomata are closed to prevent water loss and plant temperature increases as a result of lack of transpiration. Interestingly, during a combination of drought and heat stress, stomata on leaves of many different plant species are closed and leaf temperature is higher than that of plants subjected to heat alone (Rizhsky *et al*., 2002, 2004; Carmo‐Silva *et al*., 2012; Zandalinas *et al*., 2016; Cohen *et al*., 2021a). Because the temperature of reproductive processes (occurring within the flowers of cleistogamous plants) plays such a critical role in the overall yield of many crops, we studied how a combination of drought and heat stress (which has a devastating impact on yield; Mittler, 2006; Lobell *et al*., 2011; Rollins *et al*., 2013; Mahrookashani *et al*., 2017; Lawas *et al*., 2018; Liu *et al*., 2020; Cohen *et al*., 2021b; Rivero *et al*., 2022; Sinha *et al*., 2021), would impact flower stomatal aperture, transpiration and inner temperature in two different plants: soybean and tobacco (*Nicotiana tabacum*). Here, we report that during a combination of drought and heat stress, plants prioritize transpiration through flowers over transpiration through leaves by opening their flower stomata, while keeping their leaf stomata closed. This strategy, termed ‘differential transpiration’, lowers flower temperature by about 2–3°C, and represents a newly discovered acclimation mechanism of plants to different abiotic stresses that result in higher inner flower temperature (e.g. combinations of drought, pathogen infection, mechanical injury, high CO_2_ or air pollution, such as ozone, that cause stomatal closure with heat stress). Manipulating stomatal regulation, stomata size and/or stomata number (i.e. stomatal density) of flowers could therefore serve as a viable strategy to enhance the yield of different crops in the face of our uncertain current and future climates.

## Materials and Methods

### Plant material and stress treatments

Soybean seeds (*Glycine max* cv *Magellan*; United States Department of Agriculture‐Germplasm Resources Information network germplasm collection; https://npgsweb.ars‐grin.gov/) were inoculated with *Bradyrhizobium japonicum* inoculum (N‐DURE, Verdesian Life Sciences, Cary, NC, USA) and germinated in Promix HP (Premier Tech Horticulture, Quakertown, PA, USA) under short‐day growth conditions (12 h : 12 h, 28°C : 24°C, light : dark, 500 μmol photons m^−2^ s^−1^, with the temperature linearly increased from 24 to 28°C between 06:00 and 08:00 h and linearly decreased to 24°C between 16:00 and 20.00 h), for 1 wk in a growth chamber (BDR16; Conviron, Winnipeg, MB, Canada). After 1 wk, seedlings from trays were transplanted into pots containing 1 kg of Promix HP soaked in 1 l of water fertilizer (Zack’s Classic Blossom Booster 10‐30‐20; JR Peters Inc., Allentown, PA, USA) mix (Cohen *et al*., 2021a). Plants were then grown for the next 16–18 d (until the start of the first open flower, R1 developmental stage; Fehr *et al*., 1971) under the same 12 h : 12 h, 28°C : 24°C, light : dark conditions, but with the light intensity increased to 1000 μmol photons m^−2^ s^−1^. Plants were fertilized twice a week (Cohen *et al*., 2021a). At R1 plants were randomly divided into four BDR16 growth chambers placed side by side in the same room. One chamber was kept as control (CT), one as heat stress (HS), one as water deficit (WD), and one as WD + HS (Cohen *et al*., 2021a). The chambers were not randomized between experiments but were all purchased at the same time and were identical. In addition, the relative humidity was maintained at about 60–65% in all chambers, regardless of the treatment, and all internal conditions were continuously monitored. In the WD and WD + HS treatments, plants were supplied with 30% of the water available for transpiration (determined by weighing pots daily as described in Cohen *et al*., 2021a), while plants in the CT and HS treatments were well watered. The application of water deficit under these conditions mimicked ‘terminal drought’ conditions that negatively impact yield but do not kill the plant (Cohen *et al*., 2021a). Plants in the HS and WD + HS treatments were further subjected to HS by ramping the temperature from 28 to 38°C between 06:00 and 08:00 h and decreasing it to 28°C between 16:00 and 20:00 h. All measurements were conducted 10 d following the start of the stress treatments using new flowers and leaves that developed under the stress conditions (R2 stage). The temperature regime, overall temperatures, and water deficit conditions we used for our HS, WD and WD + HS treatments are comparable to field conditions in the US Midwest (Bellaloui *et al*., 2015; Cohen *et al*., 2021a,b). It should, however, be noted that the light intensity under field conditions during midday when cloud cover is at its minimum is almost double that we used in our chambers (i.e. *c*. 2000 μmol photons m^−2^ s^−1^).

### Temperature, gas exchange and water potential

Flower temperature was measured with a microthermocouple sensor (Physitemp Instruments LLC, Clifton, NJ, USA) by inserting the hypodermal needle microprobe (Physitemp Instruments LLC, Clifton, NJ, USA) 0.75–1 mm into soybean flowers (stages II and III, from R2 plants; Supporting Information Fig. S1) and 1.5–2 mm into closed tobacco flowers at 1 d before opening. Data were recorded using a Multi‐Channel Thermocouple Temperature Data Logger (TCTemp X‐Series; ThermoWorks LogMaster, American Fork, UT, USA) between 11:30 and 12:30 h. Stomatal conductance, transpiration, leaf temperature and photosynthesis were recorded using a Li‐Cor Portable Photosynthesis System (LI‐6800; Li‐Cor, Lincoln, NE, USA) between 12:00 and 13:00 h as previously described (Cohen *et al*., 2021a). Leaf temperature was also recorded using an infrared camera (FLIR C2; FLIR Systems AB, Wilsonville, OR, USA) as previously described (Zandalinas *et al*., 2020a). Water potential of leaf discs (8 mm) and flowers (cut longitudinally into half) from plants was measured using Dewpoint Potentiometer (WP4C; Meter Group Inc., Pullman, WA, USA) as described previously (Cohen *et al*., 2021a). Water potential (leaf and flowers) was measured from five to six plants (from each treatment) between 12:00 and 16:00 h.

### Measurements of stomatal aperture and stomatal density

The adaxial and abaxial surfaces of fully expanded leaves and the outer surfaces of fully expanded sepals from soybean and tobacco plants were pasted onto microscope slides, between 11:00 and 12:00 h, with a medical adhesive (Hollister Adapt 7730, Libertyville, IL, USA), as described previously (Devireddy *et al*., 2020; Zandalinas *et al*., 2020a). Stomatal aperture measurements (as a ratio of stomatal pore width to stomatal pore length) were performed for soybean and tobacco using an EVOS XL microscope (Invitrogen by Thermo Fisher Scientific, Waltham, MA, USA), as described previously (Devireddy *et al*., 2020; Zandalinas *et al*., 2020a). Both width and length of stomatal aperture were measured using ImageJ (https://imagej.nih.gov/ij). The number of stomata, epidermal and pavement cells per microscopic field of view were counted using ImageJ to calculate stomatal density and stomatal index. These were calculated as described in Ceulemans *et al*. (1995). Stomatal pore index was calculated as described in Wang *et al*. (2019). Flowers at different stages were also fixed in 4% paraformaldehyde, mounted in paraffin, sectioned and stained at the Histochemistry Diagnostic Laboratory at the University of Missouri, Columbia.

### Yield measurements

Yield and reproductive traits were measured as described in Cohen *et al*. (2021a), except that plants were scored while still growing inside the chambers, and not following recovery under glasshouse conditions. The number of flowers and pods were counted from 15 different plants per treatment, for both soybean and tobacco. Seeds from each plant (15 different plants) and seeds from individual flowers (five flowers per plants from 15 different plants per treatment) were pooled and weighed for both soybean and tobacco. Plant height was also measured at the time of yield sampling (Fig. S2).

### Abscisic acid application and sealing of stomata

Abscisic acid (ABA; Sigma‐Aldrich, St Louis, MO, USA) was dissolved in absolute ethanol, diluted to different concentrations in water (0, 50, 30, 15, and 7.5 µM) and sprayed on flowers of soybean plants (R2) growing under the different stress conditions, as previously described (Zandalinas *et al*., 2016). Control flowers were sprayed with water that contained the appropriate ethanol concentrations that matched the dilution factor (Zandalinas *et al*., 2016). Plants were then returned to the chambers and stomatal aperture was measured 60 min after ABA application. To seal stomata, petroleum jelly (Vaseline; Sigma‐Aldrich) was gently applied to flowers of plants growing under the different stress conditions using Q‐tips. Plants were then returned to the chambers and flower temperature was recorded as described above 120 min after petroleum jelly application.

### Hormone measurements

Hormone extraction and quantification were performed as previously described (Zandalinas *et al*., 2016; Balfagón *et al*., 2019). Chromatographic separation was conducted on a reverse‐phase C18 column (Gravity, 50 × 2.1 mm, 1.8 µm particle size; Macherey‐Nagel GmbH, Dueren, Germany) using a MeOH : H_2_O (both supplemented with 0.1% acetic acid) gradient at a flow rate of 300 µl min^−1^. Hormones were quantified with a TQS triple quadrupole mass spectrometer (Micromass, Manchester, UK) connected online to the output of the column through an orthogonal Z‐spray electrospray ion source. All data were acquired and processed using Mass Lynx v.4.1 software.

### RNA isolation and RT‐qPCR

Soybean flowers (stages II and III, from R2 plants; Fig. S1) were collected from plants between 11:30 and 12:30 h and immediately frozen in liquid nitrogen. For each biological repeat, 30–40 different flowers, and 15–20 different leaves, at the same developmental stage were pooled from eight to 10 different plants, and RNA was isolated using RNAeasy Plant Mini Kit (Qiagen). RNA was converted to cDNA using PrimeScript RT Master Mix (Takara, Shiga, Japan). Real‐time reverse transcription polymerase chain reaction (RT‐qPCR) was performed with gene‐specific primers (Table S1) using EF1α as internal reference using the CFX Connect Real‐Time PCR Detection System (Bio‐Rad), as previously described (Zandalinas & Mittler, 2021).

### RNA sequencing and data analysis

RNA libraries for sequencing were prepared using standard Illumina protocols and RNA sequencing was performed by Novogene Co. Ltd (Sacramento, CA, USA; https://en.novogene.com/) using NovaSeq 6000 PE150. Read quality control was performed using Trim Galore v.0.6.4 (https://www.bioinformatics.babraham.ac.uk/projects/trim_galore/) and FastQC v.0.11.9 (https://www.bioinformatics.babraham.ac.uk/projects/fastqc/). The RNA‐seq reads were aligned to the reference genome for soybean *Glycine max* v.2.1 (downloaded from ftp://ftp.ensemblgenomes.org/pub/plants/release‐51/fasta/glycine_max/dna/), using Hisat2 short read aligner (Kim *et al*., 2019). Intermediate file processing of sam to sorted bam conversion was carried out using SAMtools v.1.9 (Danecek *et al*., 2021). Transcript abundance expressed as fragments per kilobase million (FPKM) was generated using the Cufflinks tool from the Tuxedo suite (Trapnell *et al*., 2012) guided by genome annotation files downloaded from the same source. Differential gene expression analysis was performed using Cuffdiff tool (Trapnell *et al*., 2013), also from the same Tuxedo suite. Differentially expressed transcripts were defined as those that had a fold‐change with an adjusted *P* < 0.05 (negative binomial Wald test followed by Benjamini–Hochberg correction). Functional annotation and quantification of overrepresented gene ontology (GO) terms (*P* < 0.05) and KEGG pathway enrichment (*P* < 0.05) were conducted using g:profiler (Raudvere *et al*., 2019). Venn diagrams were created in Venny 2.1 (BioinfoGP, CNB‐CSIC). Venn diagram overlaps were subjected to hypergeometric testing using the R package phyper (Zandalinas *et al*., 2020a). Heat maps were generated in Morpheus (https://software.broadinstitute.org/morpheus).

### Statistical analysis

All experiments were repeated with three biological repeats, each with 15 plants as technical repeats. Results are shown as box‐and‐whisker plots with borders corresponding to the 25^th^ and 75^th^ percentiles of the data. Statistical analysis was performed using one‐way ANOVA followed by Tukey’s *post hoc* test (*P* < 0.05) in GraphPad. Different letters denote statistical significance at *P* < 0.05. Data collection for yield, stomatal aperture and stomatal index measurements was undertaken blind.

### Data availability

The analyzed transcript abundance and differentially expressed transcripts can be accessed interactively via the Differential Expression tool in SoyKB; https://soykb.org/DiffExp/diffExp.php; Joshi *et al*., 2012, 2014), a comprehensive all‐inclusive web resource for soybean. It provides a set of visualization and analytical tools such as differential expression analysis and gene card pages and provides data in the form of tabs for Gene lists, Venn diagram, Volcano plot, Function Analysis, Pathway Analysis and Gene modules.

## Results

### Leaf and flower temperature of plants subjected to a combination of water deficit and heat stress

**Fig. 1:**
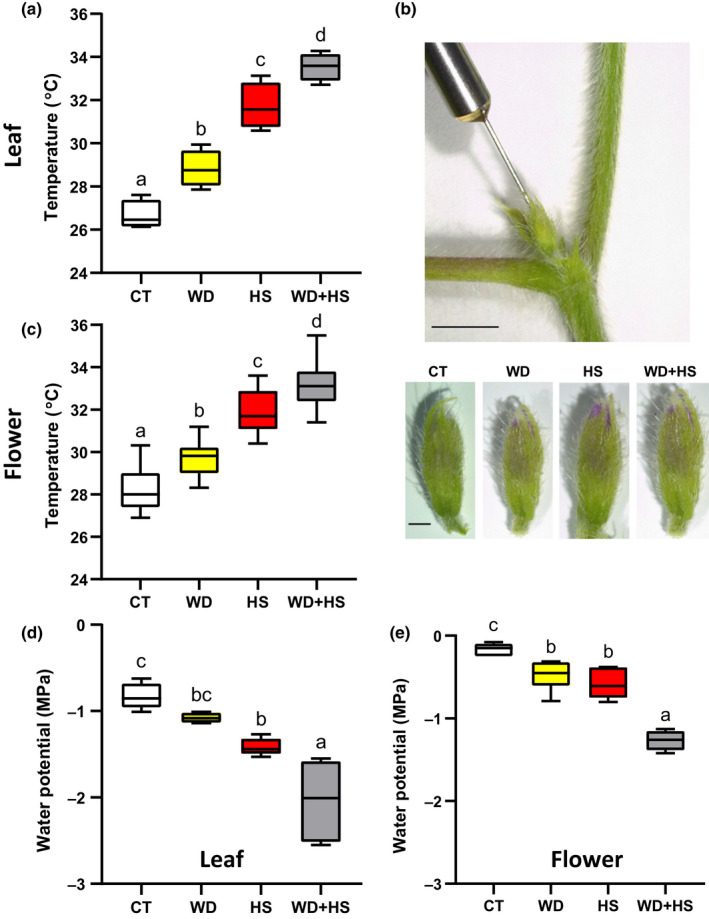
Leaf and flower temperature of soybean (*Glycine max*) plants subjected to a combination of water deficit (WD) and heat stress (HS). (a) Leaf temperature of soybean plants subjected to control (CT), HS, WD or WD + HS conditions. (b) Representative image of the experimental setup used to measure soybean inner flower temperature with a thermocouple thermometer probe (upper panel; bar, 5 mm) and representative images of closed (stages II and III; Supporting Information Fig. S1) soybean flowers developing under the different stress treatments (lower panel; bar, 1 mm). (c) Inner flower temperature of soybean flowers from plants subjected to CT, WD, HS or WD + HS. (d) Water potential (Ψ; psi, measured in MPa) of soybean leaves subjected to CT, WD, HS or WD + HS. (e) Water potential of soybean flowers from plants subjected to CT, WD, HS or WD + HS. All experiments were conducted with three biological repeats, each with 15 plants as technical repeats. Results are shown as box‐and‐whisker plots with borders corresponding to the 25^th^ and 75^th^ percentiles of the data. Different letters denote significance at *P* < 0.05 (ANOVA followed by a Tukey's *post hoc* test).

To induce conditions of WD, HS and a combination of WD and HS (WD + HS), we grew soybean plants (*Glycine max* cv *Magellan*) in controlled growth chambers. When plants began to flower (R1 stage) we induced conditions of WD, HS and WD + HS (Cohen *et al*., 2021a) and maintained these conditions for 10 d before starting to analyze and sample leaves and flowers. Using this design, we made sure that the new leaves and flowers we studied (R2 stage) developed under the different stress conditions. As shown in Fig. 1(a), as well as reported previously for different plant species (Rizhsky *et al*., 2002, 2004; Carmo‐Silva *et al*., 2012; Zandalinas *et al*., 2016; Cohen *et al*., 2021a), compared with plants subjected to CT or WD conditions, leaf temperature of plants subjected to WD + HS was higher by about 3–5°C. To determine whether flowers of plants subjected to WD + HS exhibit a similar higher temperature (compared with flowers of plants subjected to HS or WD), we measured the internal temperature of flowers using a thermocouple thermometer probe (Fig. 1b). For this analysis we used soybean flowers at stages II and III (unopened flowers undergoing self‐pollination; Fig. S1) from plants grown under WD, HS, WD + HS or CT conditions (Fig. 1b). As shown in Fig. 1(c), the inner flower temperature of flowers that developed under WD + HS combination conditions was higher by about 3–4°C than that of flowers grown under CT or WD conditions. The leaf and inner flower temperature of plants subjected to WD + HS was also significantly higher than that of plants subjected to HS (Fig. 1a,b). Water potential (Ψ; psi, measured in MPa) is typically low in tissues subjected to WD, HS or WD + HS, potentially indicating water loss and tissue dehydration (Sattar *et al*., 2020; Cohen *et al*., 2021a). In addition to increased temperature (Fig. 1a,c), the water potential of leaves (Fig. 1d) and flowers (Fig. 1e) from plants subjected to WD + HS was lower by about 0.5–1 MPa compared with that of leaves and flowers grown under CT, HS or WD conditions. Taken together, the results presented in Fig. 1 demonstrate that flowers of plants subjected to WD + HS have a high internal temperature that is accompanied by low water potential.

### Stomatal aperture and transpiration of flowers and leaves from plants subjected to a combination of WD and HS

**Fig. 2:**
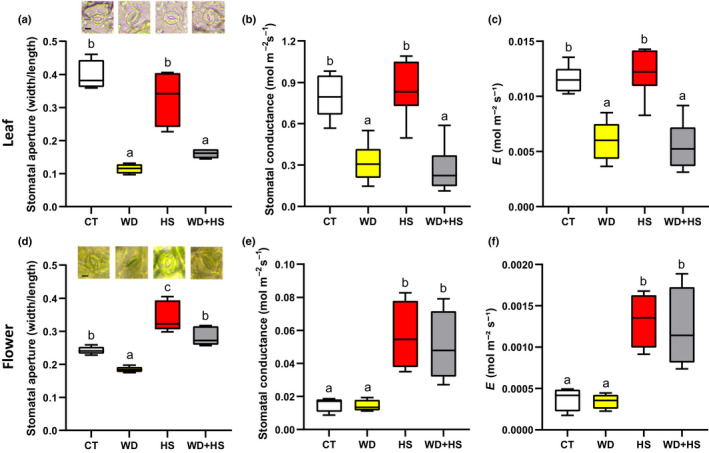
Stomatal aperture and transpiration of flowers and leaves from soybean (*Glycine max*) plants subjected to a combination of water deficit (WD) and heat stress (HS). (a–c) Stomatal aperture (a), stomatal conductance (b), and transpiration (*E*) (c) of soybean leaves from plants subjected to control (CT), HS, WD or WD + HS conditions. (d–f) Stomatal aperture (d), stomatal conductance (e), and transpiration (f) of soybean flowers from plants subjected to CT, HS, WD or WD + HS. All experiments were conducted with three biological repeats, each with 15 plants as technical repeats. Twenty microscopic fields from all parts of sepals or from the middle section of leaves (between the main veins) were measured for each plant. Results are shown as box‐and‐whisker plots with borders corresponding to the 25^th^ and 75^th^ percentiles of the data. Different letters denote significance at *P* < 0.05 (ANOVA followed by a Tukey's *post hoc* test). Representative images of stomata are shown in (a) and (d). Bar, 10 μm.

Stomatal aperture, stomatal conductance and transpiration are key physiological parameters that determine plant temperature and water potential (Nilson & Assmann, 2007; Lawson & Matthews, 2020; Hsu *et al*., 2021). We therefore measured these parameters in leaves and flowers of plants subjected to WD + HS. In agreement with our previous findings obtained with soybean, tobacco and Arabidopsis (Rizhsky *et al*., 2002, 2004; Zandalinas *et al*., 2016; Cohen *et al*., 2021a), leaf stomatal aperture, stomatal conductance and transpiration remained high in plants subjected to HS, significantly decreased in plants subjected to WD, and significantly decreased to similar values in plants subjected to WD + HS (Fig. 2a–c). These findings suggest that, in contrast to HS, leaves subjected to WD + HS could not be cooled via transpiration (Rizhsky *et al*., 2002, 2004; Mittler, 2006; Carmo‐Silva *et al*., 2012; Zandalinas *et al*., 2016; Cohen *et al*., 2021a), and experience higher temperatures (Fig. 1a). In contrast to leaves, flower (sepal) stomatal aperture and whole‐flower stomatal conductance and transpiration were significantly higher in plants subjected to WD + HS or HS than in those under CT or WD conditions (Fig. 2d,e). This finding suggests that during a combination of WD + HS, stomata of flowers (sepals) respond differently than stomata of leaves and remain open, enabling cooling via transpiration. Interestingly, the inner temperature of flowers subjected to WD + HS was high (Fig. 1c), despite the ongoing transpiration (Fig. 2f). This observation could be explained by differences in the thickness of flowers and leaves. While soybean flower buds have a diameter of about 1.5–2 mm (Fig. 1b), soybean leaves are much thinner (*c*. 0.12–0.15 mm) and can be cooled by transpiration much more easily.

### Stomatal density of leaves and flowers developed under WD + HS conditions

**Fig. 3:**
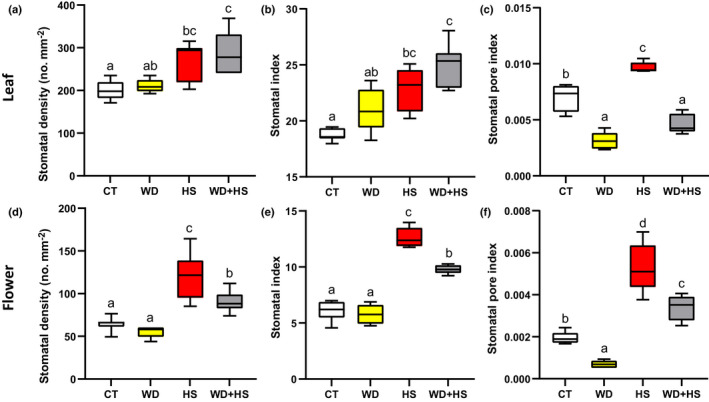
Stomatal density, index and pore index of soybean (*Glycine max*) leaves and flowers developed under conditions of water deficit (WD) and heat stress (HS) combination. (a–c) Stomatal density (a), stomatal index (b), and stomatal pore index (c) of leaves from plants subjected to control (CT), HS, WD or WD + HS. (d–f) As (a–c) but for sepals from plants subjected to CT, HS, WD or WD + HS. All experiments were conducted with three biological repeats, each with 10 plants as technical repeats. Twenty microscopic fields from all parts of sepals or from the middle section of leaves (between the main veins) were measured for each plant. Results are shown as box‐and‐whisker plots with borders corresponding to the 25^th^ and 75^th^ percentiles of the data. Different letters denote significance at *P* < 0.05 (ANOVA followed by a Tukey's *post hoc* test).

Plants display a high degree of plasticity when grown under diverse environmental conditions (Chater *et al*., 2014; Zhu, 2016; Caine *et al*., 2019; Sakoda *et al*., 2019; Lloyd & Lister, 2021; Markham & Greenham, 2021). Among the different phenotypes plants can display in response to different growth conditions is a change in the density (number per area) of stomata appearing on the surface of newly developing leaves (Chater *et al*., 2014; Caine *et al*., 2019; Sakoda *et al*., 2019). The differential responses of stomata from sepals and leaves during WD + HS, as well as the lower rates of transpiration measured from whole flowers compared with leaves (Fig. 2), prompted us to examine whether the number of stomata forming on these organs (i.e. stomatal density) during their development under the stress conditions applied in our study would also be different. As shown in Fig. 3(a,b), the stomatal density and index of leaves developed under WD + HS was significantly higher than that of leaves grown under CT conditions. Because stomata on leaves were closed under conditions of WD + HS (Fig. 2a), the stomatal pore index of leaves from plants grown under WD + HS was statistically similar to that of leaves grown under WD conditions (Fig. 3c). By contrast, while the stomatal density and index of sepals from plants subjected to HS or WD + HS was significantly higher than that of plants grown under CT or WD (Fig. 3d,e), because stomata on sepals of plants subjected to WD + HS were open (Fig. 2d), the stomatal pore index of sepals developing under HS or WD + HS was also significantly higher than that of flowers from plants grown under CT or WD conditions (Fig. 3f). The results presented in Figs 2 and 3 suggest that while the developmental responses of leaves and flowers (sepals) to WD + HS (i.e. increase in stomatal density and index; Figs 3a,b,d,e) are similar, the physiological responses of these two different organs (i.e. opening or closing of stomatal aperture; Figs 2, 3b,d) are different. It should also be noted that the expression pattern of three genes involved in the control of stomatal development on leaves (i.e. *STOMAGEN*, a positive regulator that is upregulated, and Erecta‐like1 (*ERL1*) and *ARF5/MP*, negative regulators that are downregulated; Sugano *et al*., 2010; Zhang *et al*., 2014; Qi *et al*., 2020) corresponded with the higher stomatal index and density of flowers from plants subjected to HS and WD + HS compared with CT or WD (Figs 3, S3).

### External application of ABA to flowers results in stomatal closure, and sealing of stomata results in elevated flower temperature under WD + HS conditions

**Fig. 4:**
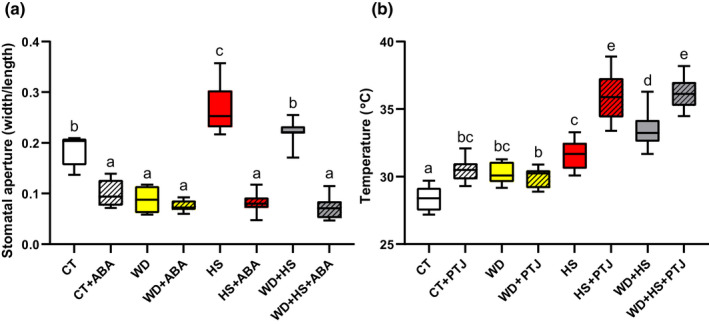
External application of abscisic acid (ABA) to soybean (*Glycine max*) flowers results in stomatal closure, and sealing of stomata results in elevated flower temperature under conditions of water deficit (WD) and heat stress (HS) combination. (a) Stomatal aperture of sepals from plants subjected to control (CT), HS, WD or WD + HS conditions at 60 min following application of 0 or 50 μM ABA. (b) Inner flower temperature of flowers from plants subjected to CT, WD, HS or WD + HS, coated or uncoated with a thin layer of petroleum jelly (PTJ) for 120 min. All experiments were conducted with three biological repeats, each with 10 plants as technical repeats. Results are shown as box‐and‐whisker plots with borders corresponding to the 25^th^ and 75^th^ percentiles of the data. Different letters denote significance at *P* < 0.05 (ANOVA followed by a Tukey's *post hoc* test).

Stomatal aperture, conductance and overall transpiration are regulated in plants by various signals (Nilson & Assmann, 2007; Buckley, 2019; Hsu *et al*., 2021). Among these, ABA is well known to play a key role in triggering stomatal closure (Nilson & Assmann, 2007; Lozano‐Juste & Cutler, 2016; Buckley, 2019; Zhang *et al*., 2022; Hsu *et al*., 2021). Because stomata of flowers from plants subjected to the WD + HS combination were open, while stomata of leaves from the same plants were closed (Fig. 2), we tested whether external application of ABA would cause stomatal closure in flowers (sepals) from plants subjected to the stress combination. As shown in Fig. 4(a), application of ABA (50 μM) to flowers grown under CT, HS or WD + HS conditions resulted in stomatal closure. By contrast, application of ABA to flowers from plants grown under WD conditions did not change stomatal aperture, as these stomata were already closed. The results presented in Fig. 4(a) suggest that stomata of flowers subjected to WD + HS can respond to external ABA application.

A possible reason why flowers would keep their sepal stomata open, maintaining transpiration under WD + HS conditions (Fig. 2), is that this process helps to lower the inner flower temperature. This could be highly important for protecting the reproductive processes occurring within the flowers of pseudocleistogamous plants such as soybean. To test whether reducing flower transpiration, by sealing stomatal apertures will cause an increase in inner flower temperature under WD + HS conditions, we used a thin layer of petroleum jelly to cover flowers (stages II and III) of plants (R2 stage) grown under CT, WD, HS and WD + HS conditions, and measured their inner flower temperature. As shown in Fig. 4(b), sealing stomatal pores with a thin petroleum jelly layer caused a significant increase of 2–3°C in inner flower temperature of flowers grown under CT, HS or WD + HS conditions. By contrast, the inner flower temperature of flowers from plants subjected to WD did not increase, as the stomata of these flowers were closed. These findings demonstrate that the opening of stomata on sepals of flowers from plants subjected to HS or WD + HS plays an important role in modulating the internal temperature of flowers, potentially mitigating some of the high temperature‐derived negative consequences for plant fertilization in cleistogamous/pseudocleistogamous plants (Rollins *et al*., 2013; Mahrookashani *et al*., 2017; Lawas *et al*., 2018; Xie *et al*., 2018; Cohen *et al*., 2021a,b; Sinha *et al*., 2021).

### RNA‐seq analysis of flower buds subjected to WD + HS

**Fig. 5:**
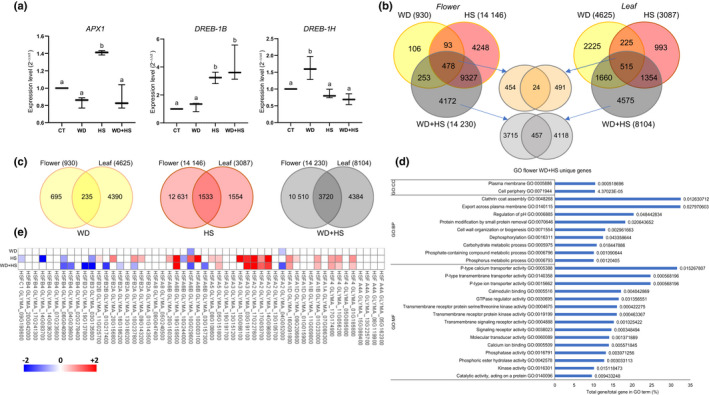
RNA‐seq analysis of soybean (*Glycine max*) flowers subjected to a combination of water deficit and heat stress. (a) Quantitative reverse transcription polymerase chain reaction (RT‐qPCR) analysis of ascorbate peroxidase 1 (*APX1*), and dehydration‐responsive element binding (*DREB*) *1B* and *1H* in flowers from plants subjected to control (CT), heat stress (HS), water deficit (WD) or WD + HS. Different letters denote significance at *P* < 0.05 (ANOVA followed by a Tukey's *post hoc* test). (b) Venn diagrams showing the overlap between transcripts with significantly altered expression (up‐ or downregulated) in flowers (left) and leaves (right) in response to HS, WD or WD + HS. Overlap between transcripts common to all stresses in leaves and flowers is shown in the upper middle and overlap between transcripts unique to WD + HS in flowers and leaves is shown in the lower middle. (c) Venn diagrams showing the overlap between transcripts with significantly altered expression (up‐ or downregulated) in flower and leaves in response to HS, WD or WD + HS. (d) Gene ontology (GO) enrichment analysis of transcripts unique to WD + HS in flowers (4177). (e) Heat map showing the expression pattern of all heat shock transcription factors (HSFs) in flowers subjected to HS, WD or WD + HS. Analysis was performed in three biological repeats. For each biological repeat 30–40 different flowers, and 15–20 different leaves, at the same developmental stage were pooled from eight to 10 different plants. All transcripts shown are significant at *P* < 0.05 (negative binomial Wald test followed by Benjamini–Hochberg correction). RT‐qPCR results are shown as box‐and‐whisker plots with borders corresponding to the 25^th^ and 75^th^ percentiles of the data. APX, ascorbate peroxidase.

To obtain a better understanding of the different processes occurring within flowers under WD + HS conditions and to compare them with the processes that occur in leaves (Cohen *et al*., 2021a), we conducted an RNA‐seq analysis of whole flowers (R2, stages II and III; Fig. 1b) collected from plants grown under CT, WD, HS, or WD + HS conditions (Datasets S1–S6). Because WD, HS and WD + HS conditions are likely to affect global processes in all tissues and cell types found in flowers, we did not dissect the flower buds into different tissues. This also allowed us to compare the RNA‐seq data obtained in the current study with a previous RNA‐seq analysis of whole leaves (that also contain multiple tissues and cell types subjected to the same conditions, reanalyzed using the same pipeline as described here; Datasets S7–S12), performed in the same growth chambers on plants from the same seed batch, under the same growth conditions (Cohen *et al*., 2021a). As shown in Fig. 5(a), RT‐qPCR analysis conducted on RNA samples before RNA‐seq analysis revealed that flowers from plants subjected to the different treatments responded differently. Transcripts encoding cytosolic ascorbate peroxidase 1 (*APX1*), a key ROS metabolizing and signaling enzyme (Davletova *et al*., 2005; Koussevitzky *et al*., 2008), significantly accumulated, for example, in response to HS, while transcripts encoding the key transcriptional regulator dehydration responsive element binding (DREB; Agarwal *et al*., 2017) *DREB‐1H* significantly accumulated during WD, and transcripts encoding *DREB‐1B* significantly accumulated during HS and WD + HS.

Venn diagrams depicting the overlap between transcripts responding to the different treatments in flowers and leaves revealed that, in contrast to leaves, flowers accumulated many more transcripts in response to HS (14 146) or WD + HS (14 230), but fewer transcripts in response to WD (930) (Fig. 5b; Datasets S13–S41). Interestingly, the numbers of transcripts with a common response to all treatments in flowers (478) and leaves (515) were very similar, suggesting that these transcripts represent a core set of WD, HS and WD + HS response transcripts. However, the overlap between these core sets of leaf and flower transcripts was low (24; Fig. 5b), demonstrating that even when it comes to the most common transcripts, the response of flower and leaf tissues to stress is different. A relatively low overlap (457) was also found between transcripts specific for a combination of WD + HS in flowers (4172) and leaves (4575) (Fig. 5b), further suggesting that the response of flowers to this stress combination is different from that of leaves. A comparison between the overall transcriptomics responses of flowers and leaves to the individual WD, HS and WD + HS treatments (930, 14 146 and 14 230 in flowers, and 4625, 3087 and 8104 in leaves, respectively) also revealed that these two tissues responded differently (overlap of 235, 1533 and 3720, respectively) (Fig. 5c). Although some overlap was found between flowers and leaves, in general there were many more flower‐specific transcripts that respond to HS and WD + HS (12 613 and 10 510, respectively), and many more leaf‐specific transcripts that responded to WD (4390). Overall, the results presented in Fig. 5(b,c) demonstrate that the response of soybean flowers to WD + HS is very different from that of leaves.

Gene ontology annotation analysis of transcripts with a unique response to WD + HS in flowers (4172; Fig. 5d) revealed that this group of transcripts is enriched in calcium signaling, kinase and protease activity, clathrin‐associated vesicle transport and other types of membrane transport mechanisms and pumps. Because different transcription factor (TF) families, such as heat shock transcription factors (HSFs), MYBs and AP2‐EREBP, play a critical role in plant acclimation to stress combination (Zandalinas *et al*., 2020b), we compared the pattern of their expression between leaves and flowers of soybean plants subjected to CT, WD, HS and WD + HS treatments (Datasets S42–S44). The pattern of expression of many of these TF families was different between flowers subjected to CT, WD, HS or WD + HS treatments (Datasets S42–S44). The pattern of expression of HSFs was for example different between flowers subjected to WD + HS, HS, or WD (Fig. 5e). This finding suggests that, compared with leaves, different types of heat and other stress responses might be activated in flowers when WD and HS are combined (i.e. WD + HS).

### Enhanced abundance of transcripts encoding the ABA degradation enzyme ABA 8′‐hydroxylase in flowers from plants subjected to HS or WD + HS

**Fig. 6:**
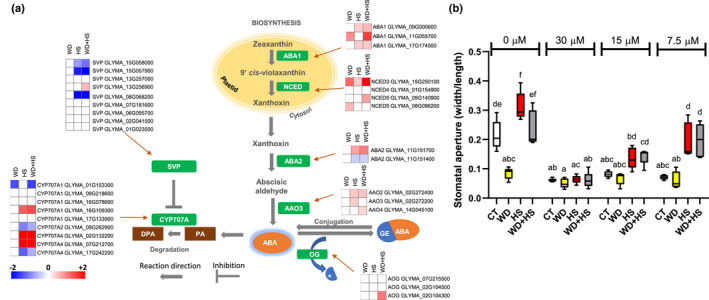
Enhanced expression of transcripts encoding the abscisic acid (ABA) degradation enzyme ABA 8′‐hydroxylase in flowers from soybean (*Glycine max*) plants subjected to heat stress (HS) or water deficit (WD) combined with HS, and higher resistance of these flowers to external ABA application. (a) Heat maps and a pathway showing the expression of transcripts involved in ABA biosynthesis and degradation in whole flowers from plants grown under control (CT), WD, HS or WD + HS conditions. All transcripts shown are significant at *P* < 0.05 (negative binomial Wald test followed by Benjamini–Hochberg correction). (b) Stomatal aperture of sepals from plants subjected to CT, HS, WD or WD + HS at 60 min following application of 30, 15, 7.5 or 0 µM ABA. All experiments were conducted with three biological repeats, each with 10 plants as technical repeats. Twenty microscopic fields from all parts of sepals were measured for each plant. Results are shown as box‐and‐whisker plots with borders corresponding to the 25^th^ and 75^th^ percentiles of the data. Different letters denote significance at *P* < 0.05 (ANOVA followed by a Tukey's *post hoc* test).

A deeper analyses of our RNA‐seq data revealed that the abundance of several transcripts encoding the key ABA biosynthetic enzymes zeaxanthin epoxidase (*ABA1*) and 9‐cis‐epoxycarotenoid dioxygenase (*NCED*) was significantly elevated in flowers from plants subjected to WD or WD + HS, while the abundance of several other key ABA biosynthetic enzymes encoding xanthoxin dehydrogenase (*ABA2*) and aldehyde oxidase (*AAO*) was significantly elevated in flowers from plants subjected to HS or WD + HS (Fig. 6a). By contrast, the abundance of several transcripts encoding the key ABA degradation enzyme ABA 8′‐hydroxylase (*CYP707A*) was specifically and significantly elevated in flowers subjected to HS or WD + HS (Fig. 6a), while the expression level of the suppressor that downregulates CYP707A (short vegetative phase; SVP; Wang *et al*., 2018) was significantly suppressed (Fig. 6a). These findings, coupled with the stomatal closure response to ABA application of sepals from flowers subjected to CT, HS or WD + HS (Fig. 4a), suggest that enhanced degradation of ABA in flowers from plants subjected to HS or WD + HS could keep ABA concentrations suppressed, and therefore stomata open under HS and WD + HS conditions (Fig. 2d).

To test whether the rate of ABA degradation is enhanced in flowers from plants subjected to HS or WD + HS, we sprayed flowers from plants grown under CT, WD, HS and WD + HS conditions with different concentrations of ABA and measured stomatal aperture (like Fig. 4a, but with lower concentrations of ABA; Fig. 6b). While higher concentrations of ABA (50 or 30 μM) caused a complete stomatal closure in flowers grown under CT, HS and WD + HS (Figs 4a, 6b), lower concentrations of ABA (i.e. 7.5, and to a lesser extent 15 μM) failed to cause a complete or significant stomatal closure in flowers from plants grown under HS or WD + HS conditions (yet caused a complete stomatal closure in flowers from CT plants; Fig. 6b). In agreement with Fig. 4(a), stomata on flowers from plants grown under WD conditions were closed and did not respond to any of the ABA concentrations applied (Fig. 6b). The findings presented in Fig. 6 suggest that the rate of ABA degradation is enhanced in flowers from plants subjected to HS or WD + HS.

### Suppressed accumulation of ABA and JA in flowers from plants subjected to HS or a combination of WD + HS

**Fig. 7:**
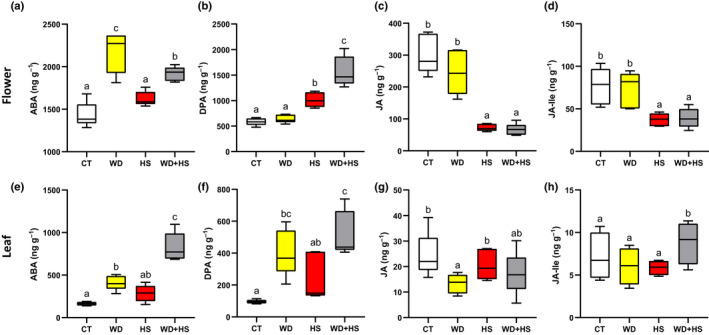
Suppressed accumulation of abscisic acid (ABA) and jasmonic acid (JA) in soybean (*Glycine max*) flowers from plants subjected to heat stress (HS) or a combination of water deficit (WD) and HS. Concentrations of ABA (a), the ABA degradation product dihydrophaseic acid (DPA) (b), JA (c), and JA‐isoleucine (JA‐Ile) (d) in flowers from plants subjected to control (CT), water deficit (WD), heat stress (HS) or WD + HS. (e–h) As (a–d), but for leaves. Experiments were conducted with three biological repeats, each with 12 plants. For each biological repeat, 30–40 different flowers and 15–20 different leaves at the same developmental stage were pooled in five technical repeats. Results are shown as box‐and‐whisker plots with borders corresponding to the 25^th^ and 75^th^ percentiles of the data. Different letters denote significance at *P* < 0.05 (ANOVA followed by a Tukey’s *post hoc* test).

The results presented in Figs 4 and 6 suggest that the rate of ABA degradation is enhanced in flowers from plants subjected to HS or WD + HS. To determine the concentrations of ABA and its degradation product dihydrophaseic acid (DPA) directly, as well as the concentrations of other hormones potentially involved in stomatal aperture regulation, we measured the concentrations of ABA, DPA, jasmonic acid (JA), JA‐isoleucine (JA‐Ile), salicylic acid (SA) and auxin (IAA) in flowers and leaves from plants subjected to CT, WD, HS and WD + HS conditions (Figs 7, S4). As previously reported for soybean, the overall concentrations of ABA were higher in flowers than in leaves (Yarrow *et al*., 1988; Liu *et al*., 2003; Wong *et al*., 2009). In agreement with our findings that transcripts encoding the ABA biosynthetic enzymes *ABA1* and *NCED* are significantly elevated in flowers in response to WD or WD + HS (Fig. 6a), the concentration of ABA in flowers from plants subjected to WD or WD + HS was significantly higher than that of plants subjected to CT or HS (Fig. 7a). By contrast, and in agreement with our findings that transcripts encoding the ABA degradation enzyme *CYP707A* are significantly and specifically elevated in flowers in response to HS or WD + HS (Fig. 6a), and that flowers of plants grown under HS or WD + HS conditions could potentially have a higher degradation rate of ABA (Fig. 6b), the concentration of DPA, a product of ABA degradation by CYP707A, was significantly elevated only in flowers from plants subjected to HS or WD + HS (Fig. 7b). These findings support our RNA‐seq analysis (Fig. 6a) and ABA application study (Fig. 6b) and demonstrate that an enhanced process of ABA degradation probably occurs in flowers from plants subjected to HS or WD + HS. Interestingly, compared with flowers from plants subjected to CT or WD stress, flowers from plants subjected to HS or WD + HS contained significantly lower concentrations of JA and the active form of JA, JA‐Ile (Fig. 7c,d). Because both ABA and JA can induce stomatal closure during stress in plants (Nilson & Assmann, 2007; Zandalinas *et al*., 2016; Zhu, 2016; Hsu *et al*., 2021; Markham & Greenham, 2021), our findings that flowers from plants subjected to HS or WD + HS contained significantly lower concentrations of JA (Fig. 7c) and JA‐Ile (Fig. 7d), as well as actively degrading ABA (Figs 6, 7b), provide a hormone‐based mechanistic understanding of the opening of stomata on flowers during HS and WD + HS (Fig. 2). In contrast to flowers (Fig. 7a–d), the concentrations of ABA, JA and JA‐Ile in leaves subjected WD + HS were not suppressed (Fig. 7e–g), and stomata on leaves of flowers subjected to WD + HS were closed (Fig. 2). Interestingly, compared with leaves from CT or WD stress, the concentration of IAA was significantly higher in leaves subjected to HS or WD + HS (Fig. S4). In addition, compared with flowers from plants subjected to WD or WD + HS, the concentration of SA was higher in flowers subjected to HS (Fig. S4; but not significantly higher than CT flowers). Further studies are needed to determine the roles of SA and IAA in plant responses to WD + HS.

### The effect of WD + HS on flower and leaf stomatal aperture, transpiration and temperature in tobacco

**Fig. 8:**
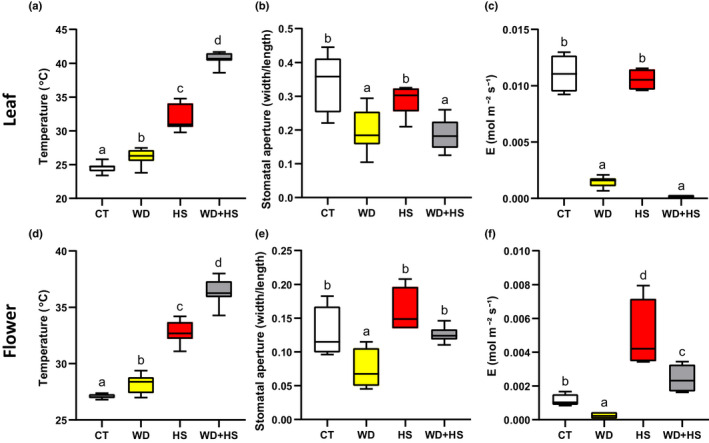
The effect of water deficit (WD) and heat stress (HS) combination on flower and leaf stomatal aperture, transpiration and temperature in tobacco (*Nicotiana tabacum*). (a) Leaf temperature of tobacco plants subjected to control (CT), HS, WD or WD + HS. (b) Stomatal aperture of tobacco leaves subjected to CT, WD, HS or WD + HS. (c) Transpiration (*E*) of tobacco leaves subjected to CT, WD, HS or WD + HS. (d–f) As (a–c), but for tobacco flowers from plants subjected to CT, WD, HS or WD + HS. All experiments were conducted with three biological repeats, each with 10 plants as technical repeats. Twenty microscopic fields from all parts of sepals or from the middle section of leaves (between the main veins) were measured for each plant. Results are shown as box‐and‐whisker plots with borders corresponding to the 25^th^ and 75^th^ percentiles of the data. Different letters denote significance at *P* < 0.05 (ANOVA followed by a Tukey's *post hoc* test).

To determine whether stomata of sepals and leaves belonging to a different plant species respond in a similar manner to soybean (Fig. 2), we studied the response of *Nicotiana tabacum* (cv SR1, *petite Havana*) plants to WD, HS and WD + HS. As shown in Fig. 8(a), and in agreement with our previous analysis of tobacco plants subjected to a combination of WD + HS (Rizhsky *et al*., 2002), the leaf temperature of plants subjected to a combination of WD + HS was significantly higher than that of plants subjected to WD or HS. This increase was accompanied by closure of stomata and suppressed transpiration (Fig. 8b,c). In contrast to leaves, and similar to soybean (Fig. 2), stomata on sepals of tobacco plants subjected to WD + HS were open, allowing transpiration to occur (Fig. 8e). Although stomata were open and transpiration occurred (Fig. 8f), the inner flower temperature of tobacco plants subjected to WD + HS (measured for unopened flowers) was significantly higher than that of flowers subjected to HS or WD (Fig. 8d; similar to our findings with soybean (Fig. 2)). As in soybean, it is possible that, owing to differences in tissue thickness between leaves and flowers, keeping transpiration ongoing in flowers is not sufficient to reduce the inner temperature of flowers more extensively.

### Yield of soybean and tobacco subjected to WD + HS

**Fig. 9:**
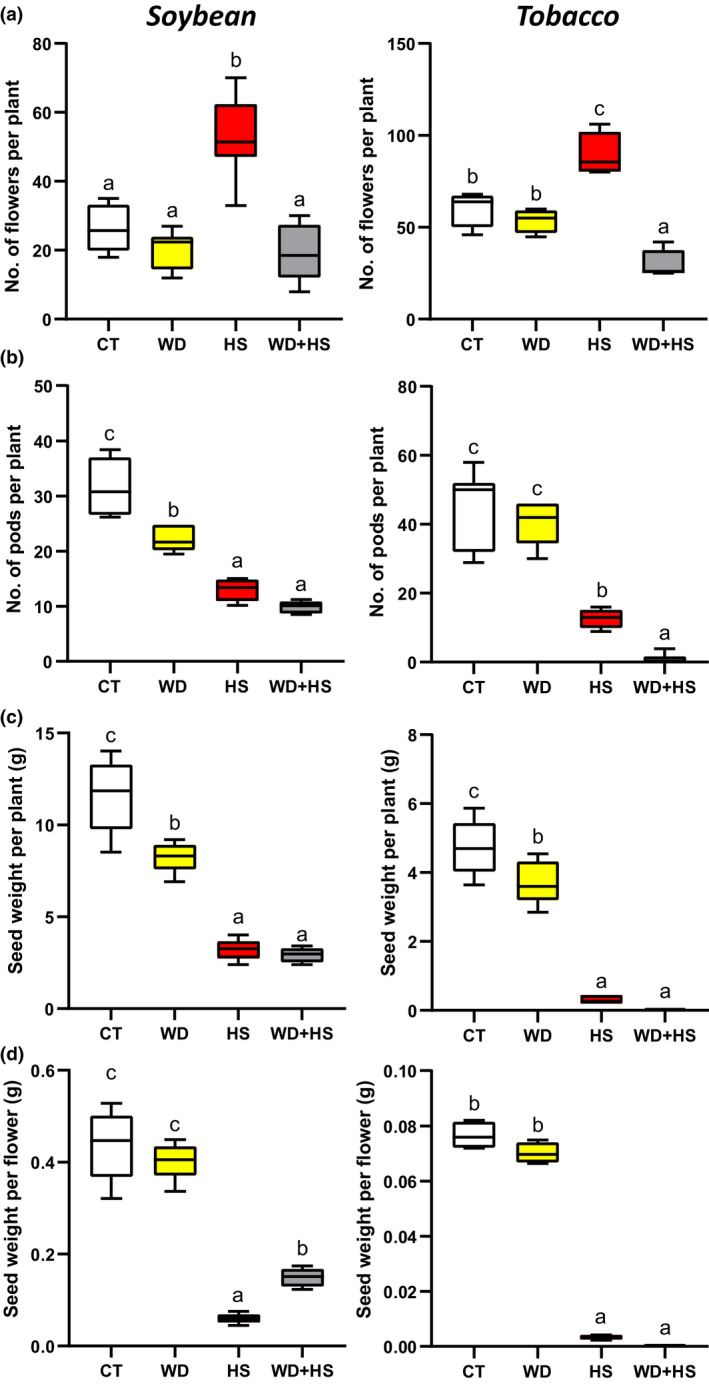
Yield of soybean (*Glycine max*) and tobacco (*Nicotiana tabacum*) plants subjected to a combination of water deficit (WD) and heat stress (HS). (a–d) Number of flowers per plant (a), number of pods per plant (b), total seed weight per plant (c), and total seed weight per flower (d) of soybean (left bar graphs) and tobacco (right bar graphs) plants subjected to control (CT), HS, WD or WD + HS. All experiments were conducted with three biological repeats, each with 10 plants as technical repeats. Results are shown as box‐and‐whisker plots with borders corresponding to the 25^th^ and 75^th^ percentiles of the data. Different letters denote significance at *P* < 0.05 (ANOVA followed by a Tukey's *post hoc* test).

Our findings that stomata of sepals are open during HS and WD + HS, and that this process limits increases in internal flower temperature (Figs 2, 4), suggest that the opening of stomata on sepals could curb the extent of yield losses that may otherwise be caused by WD + HS. Because the temperatures of flowers from plants grown under conditions of HS and WD + HS were comparable (albeit higher in plants subjected to WD + HS; Figs 1, 4, 8), we hypothesized that yield penalty in plants subjected to WD + HS will be comparable to that of plants subjected to HS alone. To test this hypothesis, we grew soybean and tobacco plants under CT, WD, HS and WD + HS conditions and scored them for number of flowers, number of pods and seed weight per plant and flower. In contrast to our previous analysis of soybean yield under these conditions (Cohen *et al*., 2021a), plants were scored for the different parameters while in the chambers, and not following a recovery period in the glasshouse. As shown in Fig. 9(a), soybean and tobacco plants subjected to HS produced significantly more flowers compared with plants subjected to CT, WD or WD + HS. The number of pods and seeds produced by plants and flowers subjected to HS was, however, significantly lower than that of plant and flowers subjected to CT or WD conditions, suggesting that most of these flowers could not produce pods and seeds (Fig. 9b–d). Interestingly, the numbers of pods and seeds produced per plant in soybean plants subjected to HS or WD + HS were comparable (Fig. 9b,c), while the number of seeds produced per flower was significantly higher in plants subjected to WD + HS vs HS (Fig. 9d). These findings suggest that the differential transpiration response of soybean plants (Figs 2, 4) could help to protect flowers during WD + HS. By contrast, HS and WD + HS had a much more severe impact on pod and seed production per plant or flower in tobacco, with WD + HS being the more severe of the two (Figs 9b–d). Our findings suggest that, at least in soybean, which uses pseudocleistogamy for plant reproduction (Takahashi *et al*., 2001; Khan *et al*., 2008; Benitez *et al*., 2010), the differential transpiration of sepals during a combination WD + HS could keep flower temperature under control and help to prevent excessive yield losses.

## Discussion

**Fig. 10:**
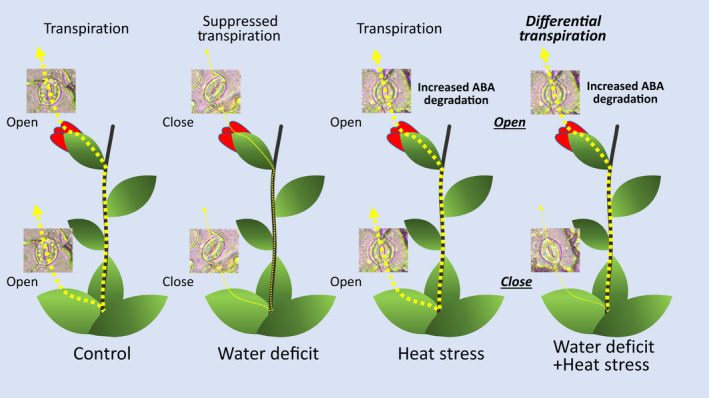
A model depicting ‘differential transpiration’ during a combination of water deficit (WD) and heat stress (HS). Control plants (left) conduct transpiration through open stomata on their leaves and flowers. In response to WD, plants (second from left) close their stomata on leaves and flowers and suppress transpiration. During HS, plants (third from left) keep their stomata on leaves and flowers open and maintain transpiration. By contrast, during a combination of WD and HS, plants (right) keep their stomata on flowers open, while closing their stomata on leaves. The opening of stomata on flowers from plants subjected to HS or a combination of WD + HS is proposed to result from an enhanced rate of abscisic acid (ABA) degradation that specifically occurs in flowers from plants grown under these conditions. The strategy of differential transpiration allows plants subjected to the stress combination to cool their flowers and limit heat‐induced negative impacts on yield.

Heat waves occurring during periods of drought can inflict heavy losses to agricultural production, especially if they occur during the reproductive growth phase of crops (Mittler, 2006; Rollins *et al*., 2013; Mazdiyasni & AghaKouchak, 2015; Mahrookashani *et al*., 2017; Lawas *et al*., 2018; Liu *et al*., 2020; Cohen *et al*., 2021b; Rivero *et al*., 2022; Sinha *et al*., 2021). Because water is needed to cool the plant via transpiration, we reasoned that when WD is combined with HS it would limit the ability of plants to cool their flowers and cause a severe heat‐induced reduction in yield. Here, we show that WD + HS conditions, which were found to reduce yield in many different crops (Mittler, 2006; Rollins *et al*., 2013; Mahrookashani *et al*., 2017; Lawas *et al*., 2018; Liu *et al*., 2020; Rivero *et al*., 2022; Sinha *et al*., 2021), are indeed accompanied by higher inner flower temperatures (Figs 1, 8). Higher leaf temperatures were previously reported for plants subjected to WD + HS and linked to the closure of stomata on leaves during stress combination (Rizhsky *et al*., 2002, 2004; Carmo‐Silva *et al*., 2012; Zandalinas *et al*., 2016; Cohen *et al*., 2021a). We therefore expected stomata of flowers from plants subjected to WD + HS to be closed as well. Surprisingly, however, they were open (Figs 2, 8). Moreover, transpiration rates of flowers from plants subjected to WD + HS were as high as those of flowers subjected to HS alone (Figs 2, 8). In contrast to flowers, stomata on leaves of plants subjected to WD + HS were closed (Figs 2, 8). Our results therefore reveal that during a combination of WD + HS annual plants prioritize transpiration through flowers over transpiration through leaves by opening their sepal stomata, while keeping their leaf stomata closed (Fig. 10). This ‘differential transpiration’ strategy lowers flower internal temperature (Fig. 4) and enables some reproductive processes to occur (Fig. 9). Under WD + HS conditions the plant might therefore attempt to protect reproductive processes, at the expense of vegetative organs. This acclimation strategy could also prove effective in other scenarios that may result in higher inner flower temperatures (e.g. combinations of pathogen infection, mechanical wounding, high CO_2_ or air pollution, such as ozone, that cause stomal closure with heat stress; Melotto *et al*., 2006; Vahisalu *et al*., 2010; Raven, 2014; Deger *et al*., 2015; Chen *et al*., 2017; Zhang *et al*., 2018; Devireddy *et al*., 2020).

Enhanced transpiration of flowers grown under WD + HS conditions could cause flowers to undergo dehydration as a result of limited water resources. Indeed, flowers of plants subjected to WD + HS had a lower water potential (Ψ) compared with flowers of plants subjected to HS alone (Fig. 2). This observation suggests that the strategy of differential transpiration under WD + HS conditions (Fig. 10) has its limits, and once flowers will reach a dehydration point of no return, reproductive processes will be further, and perhaps irreversibly, damaged. Another point to consider is the intensity of transpiration occurring from plants and whether it is sufficient to cool flowers by 2–3°C (Figs 2, 4). It is possible that other factors, as well as the cocooning effect of reproductive tissues within flowers, are playing a role in this process. The challenges faced by flowers of plants subjected to WD + HS, and the findings that they may be subjected to a combination of heat and dehydration stress (Fig. 2), is also reflected in our RNA‐seq analysis. Flowers from plants subjected to WD + HS displayed a unique transcriptomics response that was different from that of flowers from plants subjected to HS or WD (Fig. 5a,b). Interestingly, the overall transcriptomics response of flowers to WD, HS or WD + HS was different from that of leaves (with the highest degree of similarity observed between flowers and leaves subjected to a combination of WD + HS; Fig. 5c). This observation might reflect the many different reproductive processes that occur in developing flowers compared with leaves, but could also suggest that flowers are subjected to different types or degrees of stress compared with leaves under WD + HS conditions. Further studies are, of course, needed to address these intriguing possibilities. In future studies it would also be important to test whether intermittent HS or WD, or even short episodes of WD + HS, occurring during the vegetative growth stage of soybean plants, can prime the transcriptomic responses of flowers to these different stresses and their combination during the reproductive growth phase.

Our RNA‐seq analysis further revealed that the expression of several transcripts encoding the key ABA degradation enzymes ABA 8′‐hydroxylase is specifically enhanced in flowers from plants subjected to HS or WD + HS (Fig. 6a). Interestingly, stomata of flowers grown under HS or WD + HS closed in response to external application of ABA (Fig. 4a), suggesting that ABA may not accumulate to high concentrations in flowers from plants subjected to these stresses. ABA biosynthesis might therefore occur in flowers from plants subjected to WD, HS or WD + HS, but under conditions of HS and WD + HS, ABA degradation could keep ABA concentrations low and, hence, stomata open (Figs 2, 6, 7). Indeed, the concentrations of the ABA degradation product DPA were specifically and significantly elevated in flowers from plant subjected to HS or WD + HS (Fig. 7b), and lower concentrations of ABA were unable to cause complete stomatal closure in flowers from plants grown under HS or WD + HS conditions (Fig. 6b), supporting this hypothesis. In addition to enhanced ABA degradation (Figs 6b, 7b), the concentrations of JA and JA‐Ile were also specifically and significantly altered (reduced) in flowers from plants subjected to HS or WD + HS (Fig. 7c,d). Because JA‐Ile and ABA are both involved in the regulation of stomatal closure during stress (Nilson & Assmann, 2007; Zandalinas *et al*., 2016; Zhu, 2016; Zhang *et al*., 2022; Hsu *et al*., 2021; Markham & Greenham, 2021), the acclimation strategy of ‘differential transpiration’ during WD + HS (Fig. 10) could be explained by differential accumulation of these two hormones between flowers (enhanced degradation of ABA and reduced concentrations of JA‐Ile; Figs 6, 7b,d) and leaves (enhanced accumulation of ABA and JA‐Ile; Fig. 7e,h). Further studies are, of course, needed to determine how genes involved in the biosynthesis, degradation, and transport of these two hormones are differentially regulated in flowers and leaves in response to WD, HS, WD + HS and other stressful conditions.

For plants that use cleistogamy or pseudocleistogamy for reproduction, cooling of flowers by opening stomata on sepals could be especially important to protect reproductive processes from high temperatures. Although most of the closed soybean flower surface is covered by sepals (Fig. 1b), cooling of a closed flower that has a diameter of *c*. 1.5–2 mm by transpiration from sepals is much harder than cooling a leaf that has a thickness of *c*. 0.12–0.15 mm. It is likely that as a result of the thickness of flower buds, cooling by transpiration can only contribute to a reduction of *c*. 2–3°C in flower temperature during HS or WD + HS conditions (Fig. 4) and this reduction would, of course, depend on the water status of the plant. It therefore seems logical to speculate that the smaller the closed flower, the easier it will be to cool it by transpiration. Moreover, the strategy of differential transpiration, revealed by this work (Fig. 10), may be primarily beneficial for annual plants that need to produce seeds every season, as opposed to perennials that need to protect their vegetative tissues and might abort or skip flowering during entire seasons if conditions are not permissive. Because many important crops, such as soybean, wheat and barley, are annual, use cleistogamy or pseudocleistogamy for reproduction, and have relatively small flowers, the strategy of differential transpiration could play an important economic role in preventing yield penalty under different stress conditions, especially when they occur during the reproductive stage of plant growth. Further studies are needed to dissect the different pathways involved in this response and identify key regulators that control it.

The identification of differential transpiration as a potential mechanism that prevents yield losses under WD + HS conditions highlights new avenues for crop improvements. For example, the density and size of stomata on sepals or other floral organs might be altered (e.g. by manipulating the expression of different stomatal development genes; Fig. S3) to improve transpirational cooling of reproductive tissues. In addition, the pathways and mechanisms controlling stomatal responses of flowers could be manipulated to modulate opening or closing (e.g. by regulating ABA concentrations via ABA degradation; Figs 6, 7), depending on different environmental conditions, protecting flowers from overheating. These manipulations could target the timing of opening or closing as well as the different stimuli and stresses that trigger them.

In summary, our work reveals a novel acclimation strategy of plants that prioritize the transpiration of reproductive tissues over that of vegetative tissues (Fig. 10). This mechanism, termed ‘differential transpiration’, protects flowers of plants from overheating and could be important to minimize yield losses under conditions of stress combination. In addition, it can serve as a new example for plant plasticity in response to abiotic stress.

## Author contributions

RS, SIZ and YF performed experiments and analyzed the data. SS and TJ analyzed the transcriptomics data, SZ incorporated the transcriptomics data in SoyKB database, RM, FBF, RS, TJ, SS, AG‐C, SIZ and YF designed experiments, analyzed the data, and/or wrote the manuscript.

## Supporting information


**Dataset S1** Transcripts significantly upregulated in soybean flowers subjected to water deficit stress (Fig. 5b).
**Dataset S2** Transcripts significantly downregulated in soybean flowers subjected to water deficit stress (Fig. 5b).
**Dataset S3** Transcripts significantly upregulated in soybean flowers subjected to heat stress (Fig. 5b).
**Dataset S4** Transcripts significantly downregulated in soybean flowers subjected to heat stress (Fig. 5b).
**Dataset S5** Transcripts significantly upregulated in soybean flowers subjected to combination of water deficit and heat stress (Fig. 5b).
**Dataset S6** Transcripts significantly downregulated in soybean flowers subjected to combination of water deficit and heat stress (Fig. 5b).
**Dataset S7** Transcripts significantly upregulated in soybean leaves subjected to water deficit (Fig. 5b).
**Dataset S8** Transcripts significantly downregulated in soybean leaves subjected to water deficit (Fig. 5b).
**Dataset S9** Transcripts significantly upregulated in soybean leaves subjected to heat stress (Fig. 5b).
**Dataset S10** Transcripts significantly downregulated in soybean leaves subjected to heat stress (Fig. 5b).
**Dataset S11** Transcripts significantly upregulated in soybean leaves subjected to combination of water deficit and heat stress (Fig. 5b).
**Dataset S12** Transcripts significantly downregulated in soybean leaves subjected to combination of water deficit and heat stress (Fig. 5b).
**Dataset S13** Transcripts exclusively differentially expressed in soybean flowers subjected to water deficit (Fig. 5b).
**Dataset S14** Transcripts exclusively differentially expressed in soybean flower subjected to heat stress (Fig. 5b).
**Dataset S15** Transcripts exclusively differentially expressed in soybean flower subjected to combination of water deficit and heat stress (Fig. 5b).
**Dataset S16** Transcripts commonly expressed in soybean flower subjected to water deficit, and combination of water deficit and heat stress (Fig. 5b).
**Dataset S17** Transcripts commonly expressed in soybean flower subjected to water deficit stress and heat stress (Fig. 5b).
**Dataset S18** Transcripts commonly expressed in soybean flowers subjected to heat stress, and combination of water deficit and heat stress (Fig. 5b).
**Dataset S19** Transcripts commonly expressed in soybean flowers subjected to water deficit, heat stress and combination of water deficit and heat stress (Fig. 5b).
**Dataset S20** Transcripts exclusively expressed in soybean leaves subjected to water deficit (Fig. 5b).
**Dataset S21** Transcripts exclusively expressed in soybean leaves subjected to heat stress (Fig. 5b).
**Dataset S22** Transcripts exclusively expressed in soybean leaves subjected to combination of water deficit and heat stress (Fig. 5b).
**Dataset S23** Transcripts commonly expressed in soybean leaves subjected to water deficit stress and heat stress (Fig. 5b).
**Dataset S24** Transcripts commonly expressed in soybean leaves subjected to heat stress, and combination of water deficit and heat stress (Fig. 5b).
**Dataset S25** Transcripts commonly expressed in soybean leaves subjected to water deficit, and combination of water deficit and heat stress (Fig. 5b).
**Dataset S26** Transcripts commonly expressed in soybean leaves subjected to water deficit, heat stress and combination of water deficit and heat stress (Fig. 5b).
**Dataset S27** Transcripts exclusive to soybean flowers in response to water deficit, heat stress and combination of water deficit and heat stress compared with leaves (Fig. 5b).
**Dataset S28** Transcripts exclusive to soybean leaves in response to water deficit, heat stress and combination of water deficit and heat stress compared with soybean flowers (Fig. 5b).
**Dataset S29** Unique transcripts in response to combination of water deficit and heat stress exclusive to soybean flower compared with leaves (Fig. 5b).
**Dataset S30** Unique transcripts in response to combination of water deficit and heat stress exclusive to soybean leaves compared with flower (Fig. 5b).
**Dataset S31** Transcripts commonly expressed in soybean flowers and leaves when subjected to water deficit, heat stress and combination of water deficit and heat stress (Fig. 5b).
**Dataset S32** Unique transcripts in response to combination of water deficit and heat stress common between soybean flower and leaves (Fig. 5b).
**Dataset S33** Transcripts exclusively expressed in soybean flowers compared with soybean leaves when subjected to water deficit (Fig. 5c).
**Dataset S34** Transcripts exclusively expressed in soybean leaves compared with soybean flowers when subjected to water deficit (Fig. 5c).
**Dataset S35** Transcripts commonly expressed in soybean flowers and soybean leaves when subjected to water deficit (Fig. 5c).
**Dataset S36** Transcripts exclusively expressed in soybean flowers compared with soybean leaves when subjected to heat stress (Fig. 5c).
**Dataset S37** Transcripts exclusively expressed in soybean leaves compared with soybean flowers when subjected to heat stress (Fig. 5c).
**Dataset S38** Transcripts commonly expressed in soybean flowers and soybean leaves subjected to heat stress (Fig. 5c).
**Dataset S39** Transcripts exclusively expressed in soybean flowers compared with soybean leaves subjected to combination of water deficit and heat stress (Fig. 5c).
**Dataset S40** Transcripts exclusively expressed in soybean leaves compared with soybean flowers subjected to combination of water deficit and heat stress (Fig. 5c).
**Dataset S41** Transcripts commonly expressed in soybean flowers and soybean leaves subjected to combination of water deficit and heat stress (Fig. 5c).
**Dataset S42** Expression of heat shock factor (HSF) transcripts in soybean flowers and leaves subjected to water deficit, heat stress and combination of water deficit and heat stress (Fig. 5e).
**Dataset S43** Expression of MYB transcripts in soybean flowers and leaves subjected to water deficit, heat stress and combination of water deficit and heat stress.
**Dataset S44** Expression of APETALA 2 (AP2) transcripts in soybean flowers and leaves subjected to water deficit, heat stress and combination of water deficit and heat stress.

**Fig. S1** Cross‐section light microscopy analysis of fixed and embedded soybean (*Glycine max*) flowers from plants grown under controlled growth conditions.
**Fig. S2** Height of soybean (*Glycine max*) plants grown under control (CT), water deficit (WD), heat stress (HS) and WD + HS.
**Fig. S3** Expression of transcripts involved in stomatal development in flowers from soybean (*Glycine max*) plants grown under control (CT), water deficit (WD), heat stress (HS), or WD + HS conditions.
**Fig. S4** Accumulation of SA and IAA in flowers from soybean (*Glycine max*) plants subjected to heat stress or a combination of water deficit and heat stress.
**Table S1** List of primers used for RT‐PCR.
Please note: Wiley Blackwell are not responsible for the content or functionality of any Supporting Information supplied by the authors. Any queries (other than missing material) should be directed to the *New Phytologist* Central Office.

## Data Availability

Data supporting the findings of this work are provided in the main paper and Supporting Information. Raw and processed RNA‐seq data files were deposited in the GEO database (https://www.ncbi.nlm.nih.gov/geo/) (accession no. GSE186317). RNA‐seq data can also be accessed through the SoyKB database (https://soykb.org/DiffExp/diffExp.php).
