# Supplementary material for: Differential regulation of flower transpiration during abiotic stress in annual plants
Source: New Phytol. 2022 May 12;235(2):611–29. doi: 10.1111/nph.18162 (PMC9323482; doi:10.1111/nph.18162)
Supplement: Supplementary file 2 — Fig. S1 Cross‐section light microscopy analysis of fixed and embedded soybean (Glycine max) flowers from plants grown under controlled growth conditions. Fig. S2 Height of soybean (Glycine max) plants grown under control (CT), water deficit (WD), heat stress (HS) and WD + HS. Fig. S3 Expression of transcripts involved in stomatal development in flowers from soybean (Glycine max) plants grown under control (CT), water deficit (WD), heat stress (HS), or WD + HS conditions. Fig. S4 Accumulation of SA and IAA in flowers from soybean (Glycine max) plants subjected to heat stress or a combination of water deficit and heat stress. Table S1 List of primers used for RT‐PCR. Please note: Wiley Blackwell are not responsible for the content or functionality of any Supporting Information supplied by the authors. Any queries (other than missing material) should be directed to the New Phytologist Central Office. [file NPH-235-611-s001.pdf]

New Phytologist Supporting Information

Article title: Differential regulation of flower transpiration during abiotic stress in annual plants.

Authors: Sinha, Ranjita; Zandalinas , Sara; Fichman, Yosef; Sen, Sidharth ; Zeng, Shuai; Gomez-Cadenas, Aurelio; Joshi, Trupti ; Fritschi, Felix; Mittler, Ron

Article acceptance date: April 7, 2022

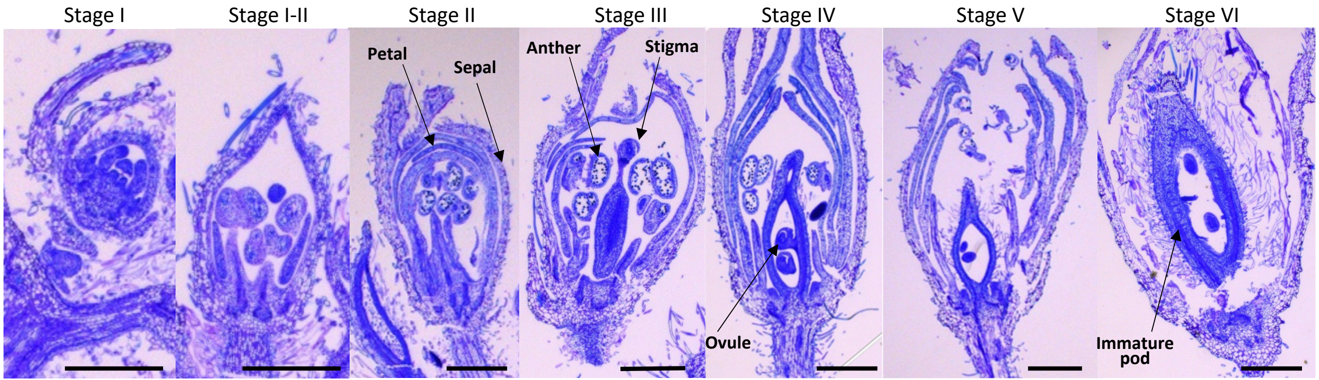

Fig. S1 Cross-section light microscopy analysis of fixed and embedded soybean (*Glycine max*) flowers from plants grown under controlled growth conditions. Bar = 500 µM.

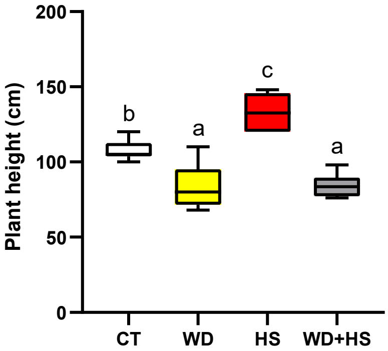

Fig. S2 Height of soybean (*Glycine max*) plants grown under control (CT), water deficit (WD), heat stress (HS), and WD+HS. Height measurements were taken 25 days post stress application. All experiments were conducted with 3 biological repeats, each with 15 plants as technical repeats. Results are shown as box-and-whisker plots with borders corresponding to the 25th and 75th percentiles of the data. Different letters denote significance at $P < 0.05$ (ANOVA followed by a Tukey's post hoc test). Abbreviations: CT, control; HS, heat stress; WD, water deficit.

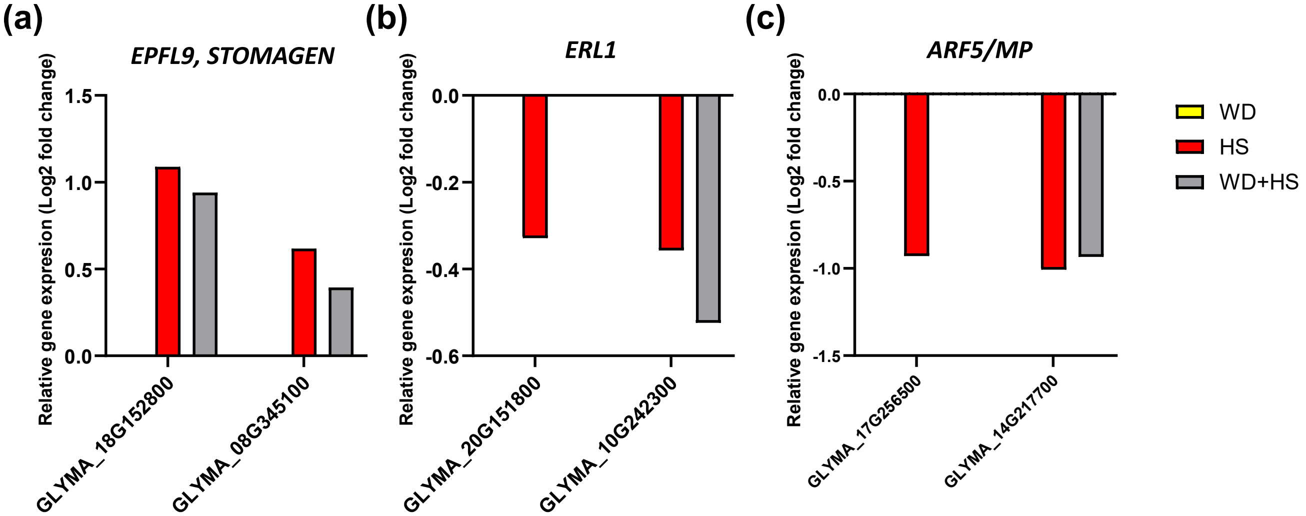

Fig. S3 Expression of transcripts involved in stomatal development in flowers from soybean (*Glycine max*) plants grown under control (CT), water deficit (WD), heat stress (HS), or WD+HS. *STOMAGEN (EPFL9)* that is upregulated (a) is known to drive a higher number of stomata, while *ERECTA-LIKE1* (*ERL1*) (b) and *ARF5/MP* (c) that are downregulated, are known to suppress the number of stomata forming on leaves (Sugano et al., 2010; Zhang et al., 2014. Qi et al., 2020). Expression level is relative to CT. Expression data was obtained from the RNA-Seq analysis.

Qi X, Yoshinari A, Bai P, Maes M, Zeng SM, Torii KU. 2020. The manifold actions of signaling peptides on subcellular dynamics of a receptor specify stomatal cell fate. Elife 9: e58097

Sugano SS, Shimada T, Imai Y, Okawa K, Tamai A, Mori M, Hara-Nishimura I. 2010. Stomagen positively regulates stomatal density in Arabidopsis. Nature 463: 241-244

Zhang JY, He SB, Li L, Yang HQ. 2014. Auxin inhibits stomatal development through MONOPTEROS repression of a mobile peptide gene STOMAGEN in mesophyll. Proc Natl Acad Sci U S A. 111: E3015-23

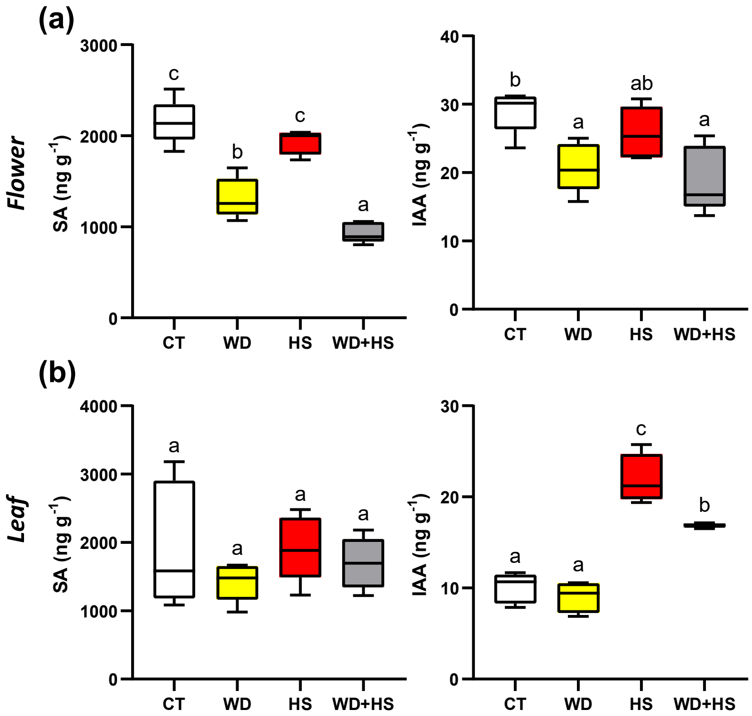

Fig. S4 Accumulation of SA and IAA in flowers (a) and leaves (b) from soybean (*Glycine max*) plants subjected to heat stress or a combination of water deficit and heat stress. All experiments were conducted with 3 biological repeats, each with 15 plants as technical repeats. Results are shown as box-and-whisker plots with borders corresponding to the 25th and 75th percentiles of the data. Different letters denote significance at $P < 0.05$ (ANOVA followed by a Tukey's post hoc test). Abbreviations: CT, control; HS, heat stress; WD, water deficit.

Supplementary Table 1: List of primers used for RT-PCR

Gene name	Forward primer	Reverse Primer
EF1-alpha	5’-GACCTTCTTCGTTTCTCGCA-3’	5’-CGAACCTCTCAATCACACGC-3’
DREB1B;1	5’- GTAAAGATTGTTCGTATGGGACAAG -3’	5’- ACACCTAAAATGAGCAACCGTACTA -3’
DREB1H;1	5’- ATTAGCCGCAAAAGTAAGTCTACG -3’	5’- TGTGCCACTAGTACTGTTGATCAC -3’
APX1	5’-TCACGGAGTTGTTGAGTGGT-3’	5’- CCTCAGCGTAATCAGCAAAG-3’
